# The Etiological Role of Blood-Brain Barrier Dysfunction in Seizure Disorders

**DOI:** 10.1155/2011/482415

**Published:** 2011-03-30

**Authors:** Nicola Marchi, William Tierney, Andreas V. Alexopoulos, Vikram Puvenna, Tiziana Granata, Damir Janigro

**Affiliations:** ^1^Department of Molecular Medicine, Cleveland Clinic Foundation, NB-20 LRI 9500 Euclid Avenue, Cleveland, OH 44195, USA; ^2^Department of Cell Biology, Cleveland Clinic Foundation, NB-20 LRI 9500 Euclid Avenue, Cleveland, OH 44195, USA; ^3^Epilepsy Center, Cleveland Clinic Foundation, NB-20 LRI 9500 Euclid Avenue, Cleveland, OH 44195, USA; ^4^Division of Child Neurology, Carlo Besta Neurological Institute, 20133 Milan, Italy; ^5^Cerebrovascular Center, Cleveland Clinic Foundation, NB-20 LRI 9500 Euclid Avenue, Cleveland, OH 44195, USA

## Abstract

A wind of change characterizes epilepsy research efforts. The traditional approach, based on a neurocentric view of seizure generation, promoted understanding of the neuronal mechanisms of seizures; this resulted in the development of potent anti-epileptic drugs (AEDs). The fact that a significant number of individuals with epilepsy still fail to respond to available AEDs restates the need for an alternative approach. Blood-brain barrier (BBB) dysfunction is an important etiological player in seizure disorders, and combination therapies utilizing an AED in conjunction with a “cerebrovascular” drug could be used to control seizures more effectively than AED therapy alone. The fact that the BBB plays an etiologic role in other neurological diseases will be discussed in the context of a more “holistic” approach to the patient with epilepsy, where comorbidity variables are also encompassed by drug therapy.

## 1. Introduction

The blood-brain barrier (BBB) is a system of capillary endothelial cells that protects the brain from harmful substances present in the blood stream, while supplying the brain with the nutrients required for proper function [1–3]. The capillary endothelium is characterized by the presence of tight junctions, lack of fenestrations, and minimal pinocytotic vesicles. In particular, tight junctions between endothelial cells form a barrier, which selectively excludes most blood-borne substances from entering the brain, protecting it from systemic influences. The BBB is anatomically and functionally associated with brain parenchymal cells. The distance between a BBB capillary and neurons is of few micrometers while the overall surface of exchange between the BBB and the brain parenchyma reaches 20 m^2^ in the adult human brain [4]. In short, the extent and complexity of the cerebrovascular interface together with the anatomical proximity of BBB vessels and neurons are highly suggestive of an active role in brain disease. In addition to the structural integrity of the BBB, there exists an enzymatic surveillance system that metabolizes drugs and other compounds bypassing the structural barrier. Recently, a strong effect of these enzymes on antiepileptic drugs (AED) metabolism has been shown in human epileptic brain [5]. 

**Figure 1:**
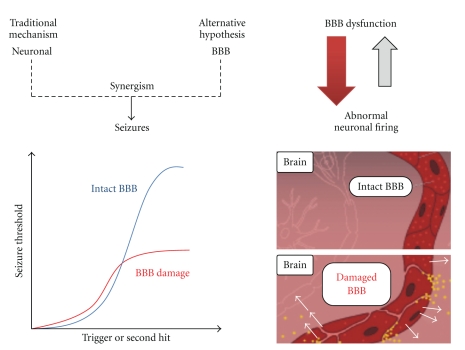
Seizure generation based on cerebrovascular events. Traditionally, the main and leading hypothesis to explain seizures consisted of abnormal neuronal wiring and excitability. While evidence of this is undisputed, the alternative/complementary hypothesis also adds to the mix pre-existing leakage of the BBB, which generates a decrease in seizure threshold. According to this hypothesis BBB leakage decreases seizure threshold independent of the fact that such leakage is associated with or a result of the seizure itself (blue versus red idealized traces). In other words, and based on the results by Friedman's Group, traumatically induced BBB disruption (BBBD) lowers seizure threshold. Others (e.g., Marchi et al.) have shown that BBBD alone is sufficient to provoke a seizure in a nonepileptic animal. While the contribution of BBB dysfunction to seizures has been demonstrated, the exact mechanisms (e.g., brain entry of peripherally circulating molecules, *yellow dots* in the cartoon) remain unclear.

Failure of the BBB has been traditionally considered the result of brain diseases (e.g., brain tumors, seizures, central nervous system infections, multiple sclerosis). As a result, the potential for a therapeutic approach to restore BBB functions has been overlooked for a more traditional neuronal take of brain pharmacology. The latter approach has been only partially successful, as evidenced by the persistent clinical burden represented by drug-resistant brain diseases [6, 7]. Most animal models of neurological disorders are based on the fact that brain neurons are the sole origin of the disorder and therefore the chief targets, while a possible role for the cerebral vasculature is often overlooked (Figure 1). 

## 2. Astrocytes and the Blood-Brain Barrier

Glial cells are numerically the predominant cell type in the brain, and the glial/neuron ratio increases dramatically with brain complexity and size [8]. Astrocytes, a specific subtype of glial cells play an important role in regulating cerebral ion homeostasis, transmitter regulation, maintenance of the blood-brain barrier (BBB), and structural, as well as metabolic, support of neuronal cells, for example, by providing the glucose-lactate shuttle [8, 9]. At the vascular level, astrocytes extend larger processes also known as “end-feet” whose terminations cover 99% of the abluminal vascular surface of capillaries, arterioles, and venules present in the cerebrovascular network. At the brain microcapillary level, these cells become one of the main building blocks of the BBB, a highly specialized dynamic and functional interface between the blood and the brain that plays a primary role in controlling and modulating the homeostasis of the central nervous system (CNS). 

## 3. Blood-Brain Barrier Function: Unless You Can Measure, You Cannot Study It

One of the problems of BBB research has been the lack of reliable methods to measure BBB intactness [10, 11]. “Opening” of the BBB provides molecules normally present in blood with open passage into the CNS. Proteins normally present in blood are free to diffuse into the CNS, and in turn, molecules and protein normally present in high concentrations in the CNS are free to diffuse into the blood. These peripheral markers of BBB opening can be detected in the blood in order to evaluate the permeability characteristics of the BBB at any given time. In brief, such markers should have low or undetectable plasma levels in normal subjects and have a higher concentration in the CSF than in plasma [10, 11]. These proteins should be normally blocked by the BBB and exhibit flux across the BBB during barrier damage. Several proteins, including S100*β*, neuron-specific enolase (NSE), and glial fibrillary acidic protein (GFAP), have been evaluated for this purpose, but only S100*β* meets all the above-mentioned characteristics [10, 11]. The fact that serum S100*β* can be used as marker of BBB integrity is not necessarily in disagreement with the notion that S100*β* is also a marker of brain damage, since both phenomena (BBB failure and brain damage) are temporally and topographically associated. In general, changes in S100*β* correlate well with radiological indexes of BBB function, such as signal changes on MRI [12, 13]. 

 These methodological aspects of BBB measurements are crucial to our understanding of the relative contribution of the BBB to seizure development. Seizures and epilepsy are commonly observed in conjunction with stroke, traumatic brain injury and CNS infections, all conditions known to result in compromised BBB function. A point of debate is whether the compromised integrity of the BBB may be a *prodromic* component of the etiology of epilepsy secondary to such pathologies (Figure 1). In support of this hypothesis is the fact that BBB damage after acute head trauma is a well-known pathologic finding in both animal and human studies. BBB disruption may persist for weeks to years after the injury and may colocalize with the area of abnormal EEG activity [14–16]. The increased interest in osmotic opening of the BBB as a viable mechanism of increased drug delivery to the brain provides an opportunity to explore the connection between BBB opening and seizures in a controlled clinical environment. Osmotic opening of the BBB by vascular infusion of a hyperosmolar bolus of mannitol is mediated by vasodilatation and shrinkage of capillary endothelial cells. Cell shrinkage results in widening of the interendothelial tight junctions to an estimated radius of 200 Å [17]. The permeability effect is largely reversed within minutes. In rodents, porcine and humans loss of BBB integrity by intra-arterial hyperosmotic mannitol has been shown to rapidly lead to EEG changes consistent with epileptic seizures [18–20], that is, spike/wave complexes interspersed with decreased EEG voltage. These studies demonstrate a correlation between the extent of acute BBB openings, as evaluated by imaging and serum S100*β* levels, and development of seizures. 

**Figure 2:**
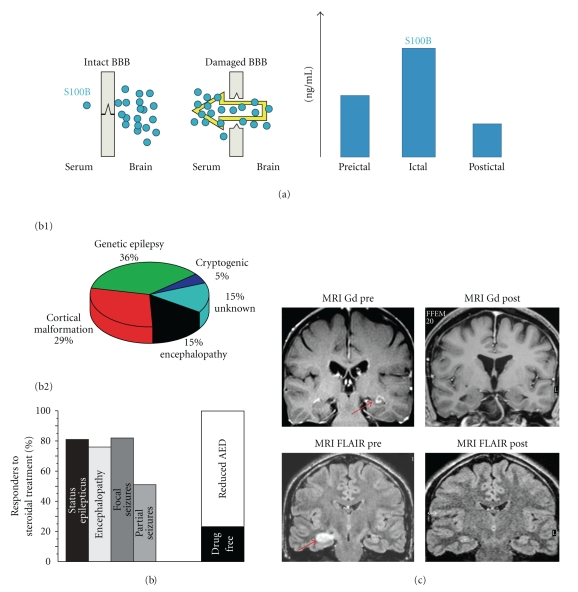
(a) Evidence in support of a link between blood-brain barrier failure and seizures in human subjects shows the rational for the use of serum S100*β* as a surrogate marker of BBBD. Extensive literature cited in this paper explains in further detail how this test is interpreted. The data presented herein refers to preliminary findings obtained in a cohort of subjects in the EEG monitoring unit. These subjects were continuously monitored for EEG changes suggestive of seizures. Serum samples were taken interictally, ictally, while another sample was taken postictally after approximately 3 hours. Note the increase of ictal S100*β* as indication of blood-brain barrier opening in these subjects. (b) Efficacy of glucocorticosteroids in a cohort of pediatric drug resistant epileptic subjects. The etiology of seizures that responded to steroids is shown in the pie chart in B1, while B2 shows the efficacy of anti-inflammatory treatment. (c) Note that discrete regions of the brain appear to have developed abnormal signal on contrast enhanced (Gd) or FLAIR sequences (see also [21]). Note that, in the MRI scans shown, the efficacy of steroids on seizures was paralleled by changes possibly associated with improved blood-brain barrier function (*n* = 2 patients).

Another example of S100*β* application is shown in Figure 2(a). We measured S100*β* serum levels to establish a temporal relation between a BBB score and seizure development. We collected blood from patients with drug-resistant epilepsy before, during, and immediately after an ictal event. Patients were continuously monitored by EEG. We found that S100*β* serum levels were elevated at the time of seizures compared to postictal, interictal levels (Figure 2(a)). The latter finding has several implications and represents the first attempt to monitor BBB status during a specific interictal-ictal-interictal transition.

A profound remodeling of the cerebral vasculature associated with leakage and extravasation of serum proteins, consequently with spontaneous seizures, is observed in rodent models of temporal lobe epilepsy. Moreover, it was recently demonstrated that angiogenesis occurs in human TLE (as well as in rodent models of TLE) as a consequence of seizures [22]. In particular upregulation of VEGF in neurons, accompanied by an increase in vascular density, has been described after acute, short- or long-lasting seizures. Once initiated, the angiogenic processes increase progressively, even in the absence of seizure activity, as observed during the latent period (e.g., in pilocarpine-treated rats), or after single short seizures induced by electro-convulsive shock. Conversely, it has also been repeatedly shown that BBB leakage promotes seizures or epileptogenesis [19, 23, 24]. Whatever the temporal relationship between BBB leakage and seizures, it is clear that the epileptic brain is characterized by an abnormal blood-brain interface (Figure 1). 

Controversial is the use of imaging techniques to detect BBB damage. The presence of brain edema can be evaluated by MRI. Specifically, structural changes at a cellular level can be assessed by diffusion-weighted imaging (DWI), which calculates the extent of passive water motion or diffusivity (apparent diffusion coefficient, ADC). Curiously, contradictory data have been obtained when evaluating the changes in brain water perfusion in rodent models and in patients with epilepsy [25–28]. DWI analysis in animal studies has demonstrated an early and transient decrease of water diffusivity during provoked status epilepticus or sustained seizures. Peri-ictal and postictal human studies, using DWI or diffusion tensor imaging (DTI), have also shown transiently decreased local diffusivity in some cases [25–28]. 

## 4. The Blood-Brain Barrier and Ictogenesis

While epilepsies affect approximately 1% of the population, seizures may occur sporadically in a much larger number of subjects [29]. Historically, a *neurocentric* philosophy has dominated the study of epilepsy and seizures, and only recently the research field has considered the fact that the cerebral vasculature is in fact intimately involved in the maintenance of proper neuronal activity and pathogenesis of seizures (Figure 1). BBB damage can occur as result of pathological events initiated “outside the brain,” such as stroke, peripheral inflammation, iatrogenic vascular manipulations, hypertension, heat, and blood hyperosmolarity. The latter are clinically associated with adverse neurological consequences such as cognitive impairment, psychiatric disturbances, and seizures. Evidence indicates that, within the periphery-brain axis, the BBB represents the key player in translating peripheral/vascular pathological events into a neuronal pathological signal, such as seizures. Perhaps the first question we must ask relates to the timing of BBB damage in relation to seizure occurrence: which comes first? Does BBB damage initiate seizures or vice versa? In experimental models of epilepsy, seizures are commonly induced by manipulation of neuronal receptors or by a kindling process. Under these conditions, neuronal death, reactive gliosis, and increased BBB permeability have been predominantly considered as the consequences of seizures [30]. This approach, and the subsequent interpretation of data, has detracted importance from the etiological role of the BBB in epilepsies and, for the most part, has impeded development of alternative pharmacological targets. Seizures are a result of a shift in the normal balance of excitation and inhibition within the brain. Given the numerous players controlling neuronal activity, it is not surprising that many different ways exist to perturb this normal balance, thus triggering seizures. Extravasation of serum albumin in the brain parenchyma was proposed as a mechanism contributing to ictogenesis and epileptogenesis in condition of damaged BBB. Direct brain exposure to serum albumin is associated with downregulation of inward-rectifying potassium (Kir 4.1) channels in astrocytes, resulting in reduced buffering capacity [31].

An important corollary of the BBB-centric hypothesis is the fact that interictal-to-ictal transitions may be caused by cycles of BBB openings. Patients with epilepsy have seizures intermittently, and, depending on the underlying cause, many patients are seizure-free for months. The sporadic appearance of seizures implies that there are *precipitating factors* inducing seizures in these patients. Seizure precipitating factors include psychological or physical stress, sleep deprivation, hormonal changes associated with the menstrual cycle, or exposure to toxic substances and certain medications [29]. All of these factors have been shown to be associated with compromised BBB permeability [30]. Severe head trauma is associated with a damaged BBB and with high risk of epilepsy [15]. The propensity of severe trauma leading to development of epilepsy suggests that brain injury results in long-lasting, pathologic changes in the brain that change a normal neural network into a hyperexcitable one. Furthermore, it is reasonable to predict that BBB damage could be sufficient to turn a “silent” brain malformation into an active one, allowing for the development of recurrent seizures and drug resistance. Pre-existing abnormalities in cortical development may contribute to the occurrence of seizures in response to a vascular challenge (*two-hit hypothesis,* Figure 1). 

## 5. Interplay between Seizures, Blood-Brain Barrier, and White Blood Cells

Experimental evidence has indicated a role of brain inflammation in epilepsy [32]. It is important to underscore that any inflammatory process, either of peripheral or brain origin, includes early vascular damage, fundamental to the propagation and maintenance of inflammation. Thus, inflammatory process follows traditional pathophysiological sequelae and is accompanied by dilation and increased permeability of blood vessels. It is surprising that, when dealing with seizure disorders, this definition is often forgotten and parenchymal cells are considered to be sole players in the inflammatory process. 

As stated above, experimental evidence supports the role of intravascular inflammation in seizure disorders. Recently, the involvement of circulating immune cells, their interaction with the BBB, and seizure propensity have been recently investigated [33]. Concordant data have been obtained using models of peripheral inflammation, such as experimental colitis, or the systemic administration of the cholinergic agonist pilocarpine [34–37]. Activation of circulating white blood cells (WBCs) was observed in animals prior to the development of seizures. In particular, pilocarpine induced acute intravascular proinflammatory changes leading to BBB leakage. In addition, loss of BBB function could be triggered by systemic proinflammatory events occurring in response to seizure activity and activation of the hypothalamic-pituitary-adrenal axis. Recently, a profound postictal change in the immune cell composition of peripheral blood in epileptics was reported [38]. In particular, NK and T CD8+ cell count was elevated. This is suggestive of the involvement of the immune axis mediated by the mesial-sympathetic connections. Based on this evidence, one may envision a model where bidirectional flux of neuroimmune information travels from and to the CNS to involve systemic organs. Departure from this equilibrium may favor seizures. 

An additional piece to the puzzle is whether or not transmigration of WBCs occurs during epileptogenesis or acute and chronic seizures. While studies have demonstrated the proseizure effect of BBB-WBCs interaction, it is not clear whether WBCs need to invade the brain to produce an epileptogenic effect. Recent evidence has provided somehow contractor results. However, it is possible that the apparent discrepancy between reports resides in the terminology used to indicate the anatomical location of cells and their quantification. For instance, WBC brain invasion was considered to occur even when a small number of WBCs (~1 cells/10 mm^2^ of brain tissue) were found in the parenchyma of epileptic human brains [33]. Our recent data showed WBC accumulation mainly at the intra- and perivascular compartments of the BBB in rodent model of seizures and brains resected from epileptic subjects [20]. Moreover, when detected in the brain parenchyma, WBC presence was limited to a specific subpopulation [39]. In particular, granulocytes appeared transiently in rat brain during epileptogenesis while monocytes/macrophages were present in the hippocampus until chronic seizures developed. B- and T-lymphocytes and NK cells were negligible [39]. The presence of brain WBC also depended on the model of seizure used. In general, it appears that a limited number of WBCs home into the brain parenchyma, while most of the WBCs are segregated to the perivascular BBB space. This is in agreement with the fact that WBC vascular extravasation under *sterile* conditions (e.g., absence of pathogens as in most of the epilepsies) is an uncommon event. The possibility also exists that WBC brain extravasation could be a reversible event. In other words, it might be that cells “extravasate” and then rapidly return into the blood stream. However, further studies are needed to rule out this possibility. Nevertheless, activated intravascular T-cells and granulocytes/monocytes can produce proinflammatory factors that, upon reaching the brain, could stimulate microglia and astrocytes causing a local inflammatory response. 

While WBC brain infiltrates are found in selected seizure disorders where a clear antigenic component is present (e.g., Rasmussen's encephalitis, [40, 41]), we now propose immunologic mechanisms of seizures applicable to a larger number of epilepsies where autoimmunity is not present. In other words, we suggest that, upon activation, WBCs act at the BBB and reside in the proximity of the vasculature without further entry into the brain parenchyma. Under these circumstances, the endpoint facilitating seizures is BBB damage regardless of subsequent WBC involvement. Whether the perivascular homing of leukocytes will lead to a more robust disruption of the BBB is possible but not yet certain. This hypothesis stems from the fact that the majority of seizure disorders are not associated with any brain immunological signature, therefore “no brain” needs to be identified and “neutralized” by the WBCs. 

## 6. Restoring Cerebrovascular Integrity to Prevent or Reduce Seizures

Given the considerations listed above, it becomes plausible that BBB repair may be of antiseizure value. If BBB damage promotes seizures, then prophylactic control of the events leading to cerebrovascular failure should be effective in preventing or reducing seizures. Preservation of BBB integrity may represent a complementary pharmacological approach to the use of neuron-targeting AEDs. Glucocorticosteroids (GCs), acting on the classic proinflammatory target and on the cerebrovasculature, may thus become clinically useful in preventing or reducing seizure occurrence (Figure 2 and [21]). 

We have recently obtained evidence supporting the effectiveness of adjunctive GCs treatment in children with intractable epilepsy; we intentionally excluded those syndromes known to be responsive to GCs and ACTH (L-G, L-K, West or Rasmussen's). GCs were beneficial regardless of the pathology and epileptic syndrome (Figure 2(c)). Similar results were obtained using the pilocarpine model of status epilepticus. We found that BBB integrity was preserved in rats pretreated with anti-inflammatory agents [21]. Preliminary results also showed that FLAIR hyperintensities were attenuated in patients who responded to CG therapy, suggesting that FLAIR is a surrogate radiologic index of BBB damage (Figure 2(b)). A comprehensive study needs to be performed in order to prove this.

The efficacy of glucocorticosteroids in reducing drug-resistant seizures remains, however, controversial. A Cochrane review suggests that steroids lack efficacy [42]. The latter study was based on a relatively small population of subjects and derived from meta-analysis of a single trial. Moreover, only ACTH was used, leaving out the use of commonly prescribed corticosteroids. In contrast, recent reports have suggested the efficacy of add-on glucocorticosteroids in pediatric forms of epilepsy [43–46]. While no conclusive studies are yet available, our recent published data [21] and preliminary data in Figure 2 provided an indication of the efficacy of glucocorticosteroids in drug-resistant pediatric seizures. We would also like to underscore that BBB damage is observed independently of the species and the type of seizures. BBB damage, as evaluated by albumin leakage, is comparable regardless of means to induce seizures [20, 23, 47, 48]. Thus, if BBB failure is a trigger of chronic as well as spontaneous, unprovoked, or iatrogenic seizures then BBB repair may impact seizure burden regardless of whether therapy is applied prophylactically or after epileptogenesis is completed. Moreover, BBB damage during epileptogenesis was found and was similar to BBB damage observed in acute animal experiments or chronic patient samples [20, 23, 47, 48]. 

## 7. Do We Need Better Experimental Models to Develop Better AEDs?

While all epilepsies are characterized by recurrent seizures, profound etiological and pathophysiological differences exist between them. These differences are often overlooked when planning laboratory experiments. Experimental models of epilepsy were originally created as drug screening tools, and a reproducible number of seizures were therefore a desirable goal. The use of these experimental models has then been expanded to the understanding of mechanisms of epileptogenesis and drug resistance. This leap has reduced a variety of clinical epileptic syndromes to a few simplistic models, disregarding the complex actuality of the epilepsies. The question remains of how to develop an appropriate experimental model able to mimic a specific epileptic syndrome. Basic research relies on models of epilepsy characterized by a rapid onset of generalized seizures, leading over time to spontaneous seizures. While these models have generated important mechanistic insights of neuronal transmission, basic science research needs to generate better models to bring the development of new therapeutic options onto a more clinically applicable level. 

There are several clinically relevant models of neonatal brain disease spanning from rodent models with genetic defects or k.o. animals, models of epigenetic inheritance, or models based on insertion of chromosomal material. Teratogen exposure (drugs and/or environmental poisons), maternal trauma, infection, and stroke are all factors that might interfere with the normal progression of brain development and give rise to aberrant patterns of cortical structure. Acquired cortical dysplasia appears to result from a progressive process (i.e., that may continue beyond the time of insult), affecting not only the primary region of lesion but also surrounding “normal” tissue [49, 50].

Malformations of cortical development (MCD) are often observed in clinical cases of drug-resistant epilepsy. Dysplastic regions are characterized by aberrant neuronal and vascular architecture. Brain regions affected by neurovascular dysplasia have a lower seizure threshold compared to normal brain [51–55]. While cortical dysplasia is a common clinical correlate of earlyonset epilepsies, it is difficult to study the basic mechanisms linking dysplastic lesions to epileptogenesis in human tissue. Models such as the methylazoxymethanol (MAM) exposed rat were until recently believed to cause MCD by a neurotoxic action. MAM is a DNA alkylating agent. Injection (i.p.) of MAM acetate into pregnant rats at day 14/15 of gestation (E14, E15) exposes the fetuses to an agent that disrupts cell proliferation at a time when neocortical and hippocampal neurons and glia are being formed [56, 57]. The most salient result of this manipulation is cortical thinning and the generation of cortical heterotopias. A number of laboratories have shown that MAM animals have lower seizure thresholds than normal controls in response to a variety of epileptogenic agents (flurothyl, hyperthermia, kindling, etc.; [58]). Studies have also suggested that these animals have behavioral impairments [59]. The main pathology that MAM recapitulates is microcephaly [60]. 

**Figure 3:**
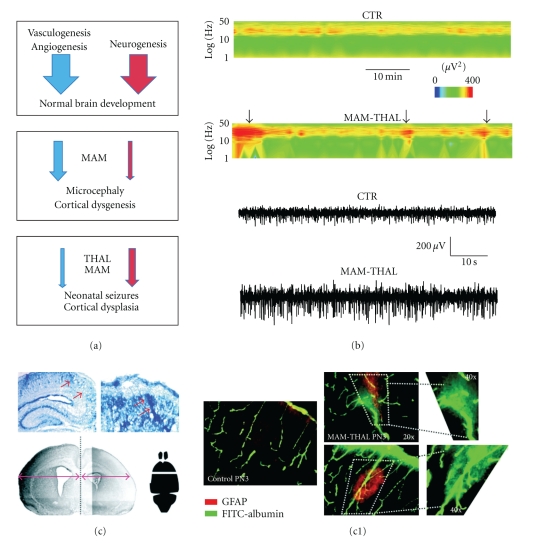
Leakage of the BBB is associated with neonatal seizures. (a) Summary of the neurovascular changes induced by prenatal exposure to MAM and/or THAL. (b) Seizures in neonatal THAL-MAM rats displayed on a joint time-frequency plot. Note also the traces recorded by the scalp electrode. (c) Loss of selective BBB permeability is associated to brain edema and hemispheric asymmetry in THAL-MAM rats. *Red arrows* point to region of disrupted cytoarchitecture due to vascular edema. (c1) FITC-albumin is used to indicate loss of BBB integrity.

Recent findings, however, have shown a remarkable toxicity of MAM towards endothelial cells and presence of dimorphic and leaky BBB vessels [56]. In this scenario the significance of *BBB damage* does not only refer to iatrogenic manipulation or traumatic events, but rather expands to various pathological changes leading to loss of fundamental BBB features, including selective permeability. In many ways, this is conceptually analogous to “membrane integrity” in cells, where small damage to membrane lipids may compromise a variety of cellular functions. Recently it has been shown that the toxins thalidomide (THAL) or MAM causes postnatal brain maldevelopment and hyperexcitability associated to abnormal vascular trunks [61] (Figure 3). In addition to seizures, prenatal exposure to THAL, valproic acid alone, or in combination with other agents [49] produces a spectrum of psychiatric and behavioral traits that are consistent with the clinical presentation of neonatal seizures and subsequent development of life-long neurological diseases. Why this occurs is not fully understood, but our previous and current results suggest that THAL and MAM, given at E15, (1) cause a transient reduction of VEGF signaling resulting in limited angiogenic potential at a time when cortical development is maximal, and (2) aborted angiogenesis results in persistence of abnormal vascular profiles [49, 61, 62], leaky BBB vessels causing brain edema at birth, increased expression of water channels, and decreased expression and function of BBB tight junctions [49, 61, 62]; (3) the combined effects of edema and BBB leakage lead to improper development and positioning of parenchymal brain cells (Figure 3), which, finally, may cause seizures and permanent brain rewiring. Remarkably, in a subset of THAL-MAM new born rats, epileptic fits were recorded (Figure 3). 

## 8. Final Remarks

The BBB has been historically studied as a “pharmacokinetic” obstacle to brain drug delivery. However, cerebrovascular failure has been recently proposed to have an etiological role in brain diseases that have been traditionally considered *neuronal in nature,* among all seizure disorders. Based on available evidence, we discussed the role of BBB failure in the initiation and sustaining of seizures and epilepsies and discussed whether a realistic clinical opportunity for BBB drugs exists. Evidence suggests that such a clinical opportunity does exist for drug-resistant forms of epilepsy, where traditional neuronal AEDs fail to control seizure, allowing for a complementary *cerebrovascular* therapeutic option.
